# Politicizing food security governance through participation: opportunities and opposition

**DOI:** 10.1007/s12571-018-0852-x

**Published:** 2018-11-26

**Authors:** Jessica Duncan, Priscilla Claeys

**Affiliations:** 1 Rural Sociology Group, Wageningen University, Wageningen, The Netherlands; 2 Centre for Agroecology, Water and Resilience (CAWR), Coventry University, Coventry, UK

**Keywords:** Civil society, Committee on world food security, Depoliticisation, Multi-stakeholder processes, Participation, Politicization

## Abstract

Since the 2007/08 food price crisis there has been a proliferation of multi-stakeholder processes (MSPs) devoted to bringing diverse perspectives together to inform and improve food security policy. While much of the literature highlights the positive contributions to be gained from an opening-up of traditionally state-led processes, there is a strong critique emerging to show that, in many instances, MSPs have de-politicizing effects. In this paper, we scrutinize MSPs in relation to de-politicization. We argue that re-building sustainable and just food systems requires alternative visions that can best be made visible through politicized policy processes. Focusing on three key conditions of politicization, we examine the UN Committee on World Food Security as a MSP where we see a process of politicization playing out through the endorsement of the ‘most-affected’ principle, which is in turn being actively contested by traditionally powerful actors. We conclude that there is a need to implement and reinforce mechanisms that deliberately politicize participation in MSPs, notably by clearly distinguishing between states and other stakeholders, as well as between categories of non-state actors.

## Introduction

Reflecting on the landscape of contemporary food security it can be concluded that efforts to ensure stable access to adequate and appropriate food for all have not succeeded. Some have gone so far as to label the longer term status of world malnutrition and hunger, and the related effects of various attempts to overcome these problems, the ‘graveyard of aspirations’ (Shaw 2007). Millennium Development Goal 1, which aimed to eradicate extreme poverty and hunger, was not met. Progress towards achieving Sustainable Development Goal 2, to end hunger, achieve food security and improved nutrition and promote sustainable agriculture, is far from optimistic.^1^ Data from 2016 suggests that the number of chronically undernourished people in the world increased to 815 million, up from 777 million in 2015 (FAO 2017). That same year, 21 countries experienced high or moderately high domestic prices for one or more staple cereal food commodities (UN 2017, 4). Aid allocated to agriculture from member countries of the Development Assistance Committee of the Organization for Economic Cooperation and Development (OECD) remains at 7 %, where it was in the late 1990s.

Food security is a dynamic concept, developed on the basis of contested knowledge and changing contexts. From a governance perspective, food security represents a policy problem for which there is no neutral diagnosis or solution: a so-called ‘wicked’ problem that transgresses traditional policy boundaries and calls for policy-making processes that reflect, orient, and include diverse experiences, knowledge and values (Rittel and Webber 1974; Termeer et al. 2015). In response, innovative modes of governance have been introduced to address this complexity, including multi-stakeholder processes (MSPs) (Breeman et al. 2015; Warner 2006). Since the 2007/08 food price crisis there has been a proliferation of MSPs devoted to bringing diverse perspectives together to inform and improve food security policy (Aubert et al. 2016). Much of the literature on MSPs highlights the positive contributions to be gained from an opening-up of traditionally state-led processes (Fischer 2000; Reed 2008; Reed 2009; Warner 2007). Yet, in there is also a convincing critique emerging to show that, in many instances, MSPs have de-politicizing effects (Kuchler 2017; McKeon 2017; Swyngedouw 2005; Westberg and Waldenström 2016), suggesting that ‘public participation has become no more than a strategy of de-politicization’ (Tsouvalis and Waterton 2011, 3).

In this paper, we provide a critical reflection on the (de)politicizing capacity of MSPs while also contributing empirically-informed insights into what happens when a policy space is politicized. In what follows we introduce the literature on MSPs followed by the theory of de-politicization. Here we pay particular attention to the critique that MSPs are a tool for de-politicization. We then identify three conditions, which are central to politicizing MSPs: common rules; a diversity of views; and, the right for everyone to speak. These three conditions guide our analysis of the most prominent food security policy space at the global level: the UN Committee on World Food Security (CFS). In line with the theory, we explore how the CFS has, since its 2009 reform, implemented conditions that enabled social movement actors representing the most affected to politicize the policy-making process. This is followed by a review of two key challenges that have emerged in response to the politicization of the CFS: a direct challenge to the ‘most-affected’ principle, and efforts by hegemonic actors to reduce political dynamics in negotiation processes through a discourse of objectivity. We conclude that while the CFS offers an emerging example of politicized participation, the politicization of the CFS could also lead to its demise. However, a just food system requires the emergence and confrontation of alternative visions that can only take place in politicized policy processes and as such, we argue the politicization of the CFS should be defended.

This paper is informed by long-term participant observation in a range of CFS activities (i.e., Plenary sessions and policy working groups) from October 2010 to February 2018, enabling us to cover the years immediately following the reform and the changes that have occurred since then. This paper also draws on a series of key-informant semi-structured interviews (*N* = 75, including follow-up interviews) conducted in English, Spanish and French between October 2016 and April 2018. Five of these participants preferred to submit written answers to open-ended questions. Informants include representatives of all different categories of CFS actors, including government representatives, CFS participants and CFS observers, selected on the basis of the key roles they play in the CFS. Interviews were transcribed and translated into English by the authors. Given the need to protect anonymity, we reference all interviewees by a relevant but non-identifying label and an interview number. Observations and interviews were triangulated with document analysis (e.g. CFS reports and policy outcomes, High-Level Panel of Expert (HLPE) reports and e-consultations, and Civil Society Mechanism documents). Key documents and interviews were first inductively coded. From there, a set of questions emerged and the documents were re-coded deductively and analyzed using Atlas.ti, a qualitative software analysis program.

## Multi-stakeholder processes (MSPs) and the (de)politicization of global food security governance

MSPs are ‘fundamentally about participatory decision-making and information sharing’ where ‘[k]ey stakeholders should be represented and decide what issues to focus on and what actions to take’ (FAO 2016). The concept of ‘stakeholders’ emerged in the 1930s to counter-balance the growing importance of ‘shareholders’ and related concerns around the responsibility of corporations to the public at large (Clarke and Stewart 1998; Lindborg 2013). The term came to be defined as ‘any group or individual that can affect or is affected by the achievement of a corporation’s purpose’ (Freeman 1984, 46). Today this includes civil society, the private sector, and even governments (FAO 2016). While form and function of MSPs vary widely, they all recognise stakeholder interests are diverse, stakes are high, and opportunities exist to impact policy (Brouwer et al. 2013).

A common criterion for identifying stakeholders in MSPs builds on the ‘all-affected’ principle (Kuchler 2017, 195). This principle implies that ‘only those who are affected by a decision should be entitled to have a say in it’ (Marchetti 2012, 31). When it comes to food security governance, this becomes challenging as everyone is affected by the organization of food systems. The universalist approach of the all-affected principle can lead to stakeholders facing different opportunities to participate (Boström and Hallström 2010, 2013; Kuchler 2017). This is because the organization of multi-stakeholder processes is an exercise in power; one that often plays out invisibly (Boström and Hallström 2013, 106). A number of authors point to the negative implications of the categorization of stakeholders when it comes to MSPs. More specifically, Kuchler (2017), focusing on the Clean Development Mechanism of the Kyoto Protocol, found that informal sorting of actors into categories of stakeholders kept civil society actors outside inner circles, and that the absence of a clear definition of stakeholder served to destabilize the distribution of participation opportunities. Echoing these findings, Rancière (1998) has argued that often efforts made at expanding participation fail to adequately include marginalized voices, prioritizing instead actors who show ‘self-discipline’. As such, many efforts at enhanced participation move towards ‘multi-stakeholderism’, effectively upholding narratives of ‘participation’ and ‘consensus’ in ways that ‘neutralize political differences’ (McKeon 2017, 385).

In this paper, we focus on growing critiques that MSPs are increasingly organized in ways that have de-politicizing effects (Clarke 2011; Fawcett and Marsh 2014; Mouffe 2005; Swyngedouw 2011). Concerns around processes of de-politicization have been raised in social science circles, and with relation to food more specifically (Duncan 2016; Moragues-Faus 2017), as part of a critique of globalization and neoliberalism and the maintenance of elite power. Swyngedouw (2005), for instance, has argued that the re-articulation of the state-civil society relationship serves to redefine and reposition the meaning of citizenship and, in turn, the nature of democracy itself. He warns that governance beyond the state can lead to encroaching of market forces, which come to set the ‘rules of the game’. Along similar lines, Lövbrand et al. (2009, 74–75) call such arrangements ‘neoliberal solutions in disguise’ noting that they can enhance problems of ‘transparency, accountability and environmental harm’ (see also Benner et al. 2004; Börzel and Risse 2005). These de-politicizing tendencies across MSPs are not to be equated with a lack of resistance or active political engagement. Indeed, ‘depoliticisation does not represent the direct removal of politics from the social and economic spheres’ (Burnham 2001, 136). Rather, de-politicization means that through MSPs, complex and normative policy processes are minimized, or structured to avoid or conceal the relations of power and conflictual dimensions inherent in them (Mouffe 1995, 262–63).

Conceptually, de-politicization encompasses a wide range of meanings (for summaries of the conceptual development see see Flinders and Wood 2014; Foster et al. 2014; Hay 2014; Wood and Flinders 2015). However, two broad yet overlapping conceptual camps can be identified from the literature: one which takes a narrow definition of de-politicization, and one which takes a more expansive conceptualization (Foster et al. 2014, 227). The narrow definitions see de-politicization primarily as a tool, mostly of governments. This approach considers de-politicization as a set of activities that seek to limit or remove the political domain from the public sphere. Examples include having technical teams define political objectives (e.g. indicators and targets of the Sustainable Development Goals). It also includes activities that replace the communicative rationality of the political domain with another rationality. For example, scientific rationality is often adopted by technocrats who resist disagreement by characterizing it as ignorance or ideological.

The expansive definitions of de-politicization look at the broader processes that may limit the availability of spaces where the political can play out; where political agency can occur. This includes the implementation of processes that seek to replace disagreement and a lack of consensus with consensus among so-called disciplined stakeholders (i.e., those willing to play the game) who are invested in avoiding being labelled as ‘extremists’ (Swyngedouw 2009; Walters 2004). Those who apply these definitions are centrally concerned with the relationship between processes of de-politicization and politicization (Foster et al. 2014, 227). In this paper we apply an expansive definition of de-politicization as a process.

In terms of assessing the politicization of the CFS, we draw from Rancière (1998, x–xii) who perceives political deliberation – the antidote to de-politicization – to be founded on disagreement. In this context disagreement is not meant to denote general misunderstandings but refers instead to making visible unequal relations of power within consensus-driven policy spaces. That is, disagreement must be over the very nature of the situation itself: about the assumed arrangement of things (Rancière 1998). This points to disagreement being based on different, often competing, worldviews, one of which will be hegemonic and thus upheld by traditional elites. Given this, it is not simply disagreement that is a key condition for politicizing, but also ensuring that there is space for disagreements which highlight competing experiences and understandings of the problem while simultaneously recognizing the relations of power associated with each worldview. Following the theory, disagreements of this nature are unavoidable and even necessary since they are a representation of the varied global society (Mouffe 2005). Only by creating spaces where these fundamental disagreements can be articulated can we start to find shared meanings and ultimately design global policies in which a broader range of the global population can benefit (Clark et al. 1996).

Towards this end, Rancière (1998) argues that to re-politicize policy spaces, actors need to: a) agree to a common set of rules of engagement; b) ensure a diversity of views are represented; and, c) ensure everyone, including ‘extremists’, have the right to speak. Recognizing that these conditions are overlapping and interconnected, in what follows we use them to guide our analysis of processes of politicization across the Committee on World Food Security (CFS). In the next section, we look at the common set of rules that instituted the CFS as an MSP following the 2009 CFS reform. We then turn to the second condition and discuss the mechanisms that enable a diversity of views to be represented through categorizing the participation of different non-state actors within the CFS. Our exploration into the third condition looks at how the ‘extremists’, who in the case of the CFS would be the social movements representing the most affected, have self-organized through the Civil Society Mechanism (CSM).

### Reforming the CFS: Establishing a set of common rules

In October 2009, 101 member-country delegates met at the headquarters of the United Nation’s (UN) Food and Agriculture Organization (FAO) to approve reforms to the UN’s Committee on World Food Security (CFS) so that the Committee could ‘fully play its vital role in the area of food security and nutrition, including international coordination’ (FAO 2013, 207). Through the reform the CFS declared itself to be a ‘multi-stakeholder platform that enables all viewpoints to be considered’ (CFS 2011). The reform, along with the CFS Rules of Procedure, represent the set of common rules of engagement that actors agree to when they enter the CFS policy space. In what follows, we explain the rationale and structure of the reformed CFS, with particular consideration to the common rules that inform two of the key pillars of the reform: inclusivity through participation and evidence-based policy outcomes.

The reformed document of the CFS outlines clear rules for participation organized around three categories: Members, Participants and Observers. Membership is open to all member states of the United Nations. Observers are other entities and individuals (such as academics) who can request to be invited to observe entire sessions or specific agenda items. Participants are non-state actors, specifically civil society actors, the private sector, philanthropic foundations, financial institutions, international research bodies, and UN institutions. Member states are to ‘take into consideration the views of all participants and stakeholders to the fullest extent possible in order to foster ownership and full participation’ (CFS 2009, para. 18).

**Fig. 1:**
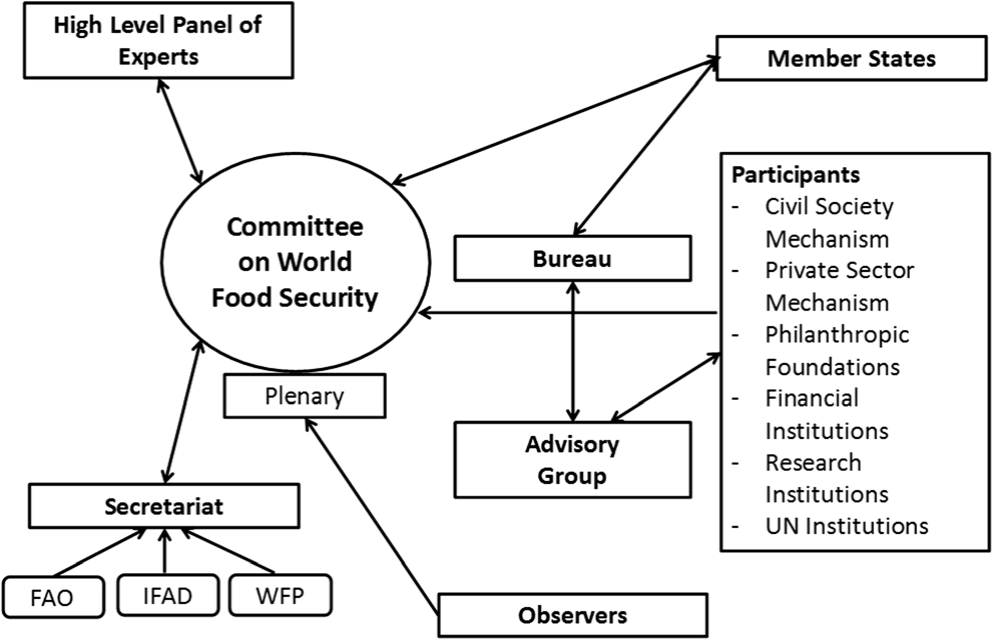
Structure of the Committee on World Food Security. Source: Duncan 2016

The CFS includes a number of structures where members and participants interact (Fig. 1). The Bureau, made up of only member states, is the executive arm of the CFS and is supported by the Advisory Group, made up of representatives from the participant categories. Intersessional work is organized around Technical Task Teams and Open-Ended Working Groups made up of member states and participants. The Plenary is the central body for decision-taking, debate, coordination, lesson-learning and convergence by all stakeholders at a global level on food security issues. Plenary sessions are held annually and attended by members, participants and observers. The CFS has a permanent Secretariat made up of staff from three Rome-based UN agencies: FAO, IFAD and WFP. It works to support the Plenary, the Bureau and Advisory Group and the High-Level Panel of Experts (HLPE). The Secretariat is currently hosted at FAO in Rome Fig. 1.

In terms of decision-making, the CFS has formal rules but also makes use of non-formalized procedures. In practice, when developing policy outcomes, the CFS aims to achieve consensus amongst all members and participants. When consensus is not possible amongst these actors, consensus is sought from member states. In the formal CFS rules and procedures, states maintain the right to vote and voting could be used if consensus is not found. At the time of the reform, one of the arguments put forward by supporters of the CFS, notably the FAO, the Latin America government regional grouping and civil society organizations (CSOs), was the commitment to the principle of ‘one country, one vote’ (Duncan 2015, 70). With regard to politicization, the fact that the 2009 CFS reform maintained accountability with states runs counter the neoliberal tendency to reduce the role of states – the so-called hollowing-out of the public sphere (Flinders and Wood 2014, 137). Proponents of the CFS argued that one-country, one vote served to even out geo-political power imbalances, which would only be reinforced through for example, G8- or G20-led initiatives that were competing with the CFS for influence at the time. Because of the commitment to one country, one vote, weaker states can play an important role and have had important influence in shaping policy outcomes (Duncan 2015, 216). This has been further supported by the presence of participants, particularly civil society actors in the negotiations, enabling states and participants to create alliances. As a result, some traditionally weaker countries have been able to push forward or support issues that go against the status quo (Duncan 2015, 216).

Addressing politically contentious issues is fundamental to identifying appropriate solutions for rebuilding food systems (i.e. access to land and natural resources, right to water, women’s rights, food sovereignty and access to local or territorial markets). Within the unique participatory space of the CFS, the commitment to achieve consensus raises interesting tensions when it comes to theories of de-politicization. Following these theories, consensus-based processes have been actively critiqued for eliminating the voices of those who do not follow the status quo, and for masking apathy (Mouffe 1993, 2005; Swyngedouw 2009; Walters 2004). Related to this, a central strategy of de-politicization is to make use of consensus processes to avoid the discussion of contentious issues: that is to limit disagreement. As such, in many governance processes, contentious, conflictual and difficult issues are set aside in favour of issues on which actors can reach consensus (Coglianese 1999). However, as we elaborate below, in the CFS the commitment to finding consensus among states and autonomous participant groups has had the opposite effect and the inclusion of participants across the work-streams of the CFS has established clear pathways for the introduction of contentious issues onto the CFS agenda (Duncan 2016).

To summarise the first condition for politicizing participation, within the reformed CFS there has been agreement around common rules, and importantly, these make space for the next two conditions for politicization in that they set out criteria for the inclusion of a diversity of perspectives. The rules of engagement at the CFS challenge the idea that consensus-based decision making is necessarily depoliticizing by ensuring active engagement of a diversity of participants but also by placing ultimate decision-making power in the hands of states (in case there is no consensus). Further, the CFS reform upholds the principle of one-country one-vote, which gives traditionally weaker states a stronger voice and supports alliances that might normally be overlooked. At the same time, these rules reinforce the key role of states, making them accountable to address food insecurity and implement the right to food, which is at the heart of the CFS mandate.

### Participants with intervention right: Ensuring a diversity of views are represented

As noted above, the CFS has declared itself to be a ‘multi-stakeholder platform that enables all viewpoints to be considered’ (CFS 2011). One of the most significant elements of the reform was the allocation of participation rights to non-state actors. By way of reform, and through the inclusion of participants, the CFS agreed to ‘seek to achieve a balance between inclusiveness and effectiveness’ (CFS 2009, 7).

Rather than fall prey to an ambiguous categorization of stakeholders, the CFS reform structure seeks out a diversity of participants, while explicitly prioritizing those most affected (discussed in detail in the next section). This has been backed up by formal opportunities for participants to shape policy processes and outcomes. The formalization of such high-level participation is unprecedented within the UN system and food governance fora beyond the UN system. The official designation of ‘participants’ allows these actors to ‘take part in the work of the Committee with the right to intervene in Plenary and breakout discussions to contribute to preparation of meeting documents and agendas, submit and present documents and formal proposals’ (CFS 2009, para. 12). This move was significant. As one civil society actor explained:

the change of our status from observers to participant was a unique change. (…) I think we have really utilized that space at the beginning and also could create some of the good outcomes based on that since we have also this equal footing thing with the governments on our opinions (Interview 34).

To facilitate participation in the work of the CFS, civil society actors and the private sector both created their own autonomous and independent mechanisms. These are the only two categories of participants to have developed such mechanisms. For UN Agencies, philanthropic foundations and research institutes, specific organizations have been identified as participants, for example the Gates Foundation and the global agricultural innovation network CGIAR.

For civil society, there is the autonomous International Food Security and Nutrition Civil Society Mechanism (CSM). Actors who participate in the CSM have organized themselves around seventeen sub-regions and eleven constituencies: smallholder farmers, pastoralists, fisherfolk, indigenous peoples, agricultural and food workers, landless, women, youth, consumers, urban food insecure and NGOs. Within the governing body of the CSM, the Coordination Committee, quotas are used to ensure balance between constituencies, sub-regions and gender (Claeys and Duncan 2018a). Members of the Coordination Committee are elected by way of autonomous processes. While recognizing International NGOs as one of its constituencies, the CSM makes a clear distinction between NGOs and social movements and prioritizes social movements’ voices (this distinction is elaborated upon below). The CSM is coordinated by a Secretariat based in Rome.

Like the CSM, the private sector established a mechanism to facilitate participation of private enterprises across the agri-food value chain in the work of the CFS.^2^ The Private Sector Mechanism (PSM) is led by the International Agri-Food Network (IAFN) and is open to all those involved in addressing agriculture, food security and nutrition from a business point of view – including farmers, input providers, cooperatives, processors, small and medium sized enterprises (SMEs) and food companies (PSM 2017). It is important to note that at the time of the reform (2009), the CFS was not garnering a lot of interested from the private sector. As a result, the PSM has been much slower in developing than the CSM. It has however grown in size since the reform and is now very active in policy processes. In 2010, in the first post-CFS session, the only participant from the private sector representative was Croplife International (CFS 2010). In 2017, the PSM included representatives from 58 private companies (CFS 2017, para. 1), including Bayer Crop Science, Cargill Europe, Ethanol Europe, Monsanto, Nestlé and Syngenta. Unlike the CSM which receives institutional funding, the PSM offers three types of memberships, linked to a fee structure: supporting members (€12,000); contributors (€2500); and non-paying members. Like the CSM, the PSM organizes around thematic working groups following the CFS’s work streams.

These two mechanisms are fundamental to facilitating the engagement of diverse voices into the policy process. As one state representative noted:

The added value [of the CFS] is the unique nature of this Committee. This is really the challenge but also the advantage of the Committee. That you have much more difficult discussions, of course, together with the stakeholders. It’s much more difficult to discuss it, especially because the two big mechanisms, CSM and PSM, have very different views on specific topics, but at the same time, it’s also a big advantage because the product and also the outcomes in general of the Committee has much more link to the reality, so to say (Interview 57).

In the CFS, policy outcomes are initially informed by reports requested to the High-Level Panel of Experts (HLPE). The HLPE is the science-policy interface of the CFS. It is made up of a Steering Committing of internationally recognised experts in food security and nutrition-related fields as well as ad hoc project teams comprised of experts working on a project-specific basis. The result is a ‘consortium of heterogeneous knowledge and experiences not limited to the usual fields of formal research’ (Colombo and Onorati 2013, 71). The HLPE is tasked with providing ‘scientific and knowledge-based analysis and advice on specific policy-relevant issues, utilizing high quality research, data and technical studies’ and identifying ‘emerging issues’ and helping ‘members prioritize future actions and attentions on key focal areas’ (CFS 2009). The ability of the HLPE to include non-published sources in its reports represents an important step towards broadening the scope of evidence and knowledge that informs food security and nutrition policy. As the Rules and Procedures of the HLPE state:

Non-published sources, reporting of field projects, or other non peer-reviewed sources are accepted as relevant information sources, as far as their content is accessible to the HLPE and their quality is reviewed by the project team before incorporation in the HLPE report (HLPE 2010, 25).

At the same time, this almost inevitably leads to the collection of data that is contradictory, or even conflictual. Yet, the HLPE is not meant to smooth over, select sides or seek compromise, rather its job is to synthesize. As the first chairman of the HLPE, M.S. Swaminathan, stated ‘[o]ne of the key roles of the reports is to help members and participants in CFS to understand why they disagree’ (HLPE 2017b, 2). This commitment to not only accepting disagreement, but supporting enhanced understanding of this disagreement, is central to politicizing a policy-making process. Towards this end, the HLPE serves a political function insofar as it expands the scope of knowledge and expertise beyond traditional categories, challenging the de-politicizing tendencies often associated with discourses of evidence-based decision making. Indeed, the selection of what evidence should inform policy is not a neutral process and research has shown that decision-makers often hide the political nature of decision-making behind claims of objectivity (Duncan 2016).

This section has shown how the CFS reform makes space for a diversity of views to not only be represented, but also heard. Agreeing to work towards consensus on contentious issues in a context of expanded formal interventional rights to clearly defined categories of stakeholders is a key condition for politicizing an MSP. The expansion of this diversity of views and forms of knowledge beyond policy negotiations into the scientific body of the CFS, the HLPE, works to further politicize the policy process.

### Everyone has the right to speak: A focus on those most affected

As noted above, a common criterion for identifying stakeholders in MSPs builds on the ‘all-affected’ principle (Kuchler 2017, 195), meaning that all those potentially affected by a decision should be entitled to have a say in it. In the case of the CFS, rather than the all-affected principle, the reform process advanced what we could call a ‘most-affected’ principle, giving priority voice in the policy-making process to those most affected by hunger and food insecurity. As written in the reform document, the composition of the CFS will ‘ensure that the voices of all relevant stakeholders – *particularly those most affected by food insecurity* - are heard’ (CFS 2009, para. 7 emphasis added). In this way, rather than trying to level the so-called playing field, the CFS set out to account explicitly for different experiences and power relations between and across participant categories. Particularly relevant towards this end is the list of categories of people to receive additional attention. The CFS reform document states that the Civil Society Mechanism will ‘also serve inter-sessional global, regional and national actions in which organizations of those sectors of the population most affected by food insecurity, would be accorded priority representation’ (CFS 2009, 16).

The added value of prioritizing the voices of those most affected by food insecurity in policy-making is well recognized by certain member states. As one state representative noted:

The experience of the most affected, I think, from food insecurity and malnutrition because from the smallholders and the producers of food and it’s very important to hear their perspective. Because in the vision of CFS we have the words ‘the most affected by food insecurity’ and they are the voice of these most affected. This is the most important thing they bring to CFS because sometimes we could lose the central point of what we are doing (Interview 56).

This point of view is echoed by another state representative who commented:

It’s not only governments negotiating it. It’s really people who are most affected by hunger and malnutrition, and also by companies directly, who are engaged in the Plenary, and that, of course, is a very good momentum for everything that comes out of the Committee. It gives more value to the product (Interview 57).

The reform principle of prioritizing the voices of those most affected has been anchored in the governance structure of the CFS, and explicitly translated into specific CFS processes, although it could still be strengthened. At the heart of these processes is the agreement that the CFS would include a variety of different civil society constituencies – e.g., small-holder farmers, fishers, Indigenous Peoples, pastoralists, agricultural workers, NGOs, etc. – (Claeys and Duncan 2018a), and seek to achieve gender and geographic balance in civil society representation (CFS 2009, para. 11). To facilitate the participation of civil society organizations representing the most affected, the CSM has secured specific institutional resources enabling civil society representatives to travel to Rome, and access the translation and interpretation services they need to speak on their own behalf. Most importantly, the CSM has internally organized in ways that give leadership roles to social movement representatives (Claeys and Duncan 2018a) (see below). In addition, the CSM was granted more seats on the Advisory Group to the CFS Bureau than other participants: The CSM has four seats, while the other participant categories only have one seat each.^3^ This decision was justified not only by the argument that civil society actors represent those most affected by food insecurity, but also in recognition of the diversity of actors across civil society. Finally, the CFS commitment to prioritizing the voices of those most affected is expressed through a number of informal, but widely used, practices such as: the allocation of speaking time to CSM participating organizations in Plenary, the choice of keynote speakers from the CSM, and the selection and training of Technical Task Team coordinators coming from the CSM.

One key factor that distinguishes the CFS from most other MSPs should be highlighted here. It is that civil society actors actively politicize the CFS because they have organized amongst themselves through the CSM in highly political ways. Reflecting back on the issue of categorization, civil society actors have implemented categories of participation that explicitly prioritize the voices of social movements vis-à-vis those of NGOs. CSM policy working groups, for example, are all led by one or more social movement representative(s), supported by a technical facilitator coming from an allied NGO. Social movement actors (such as La Via Campesina, or the World Forum of Fish Harvesters & Fish Workers) bring in demands, experiences and perspectives that provoke disagreement within the CFS and in turn, force the Committee to move beyond status quo. The politicization of participation in the CFS thus goes beyond the fact that participants are able to autonomously organize. It relates to the fact that the CSM space is occupied by social movement leaders (Claeys and Duncan 2018b). This shows that while rules and mechanisms are key to supporting politicization, the political agency of actors also plays a fundamental role.

Post-reform, the active organization and participation of the CSM in the CFS, as well as its strong presence on the Advisory Group, helped the CSM establish a balance of power that was favorable to civil society actors, and social movements in particular. As one CSM actor involved since the 2009 reform explained:

With regards to the voices of the most vulnerable, this is also a crucial thing that we have achieved. We were able to make a collective voice and to make our voice more stronger than before as individual organization. That was also a positive impact (Interview 34).

However, as we discuss below, all of these practices are questioned or targeted by some CFS members or participants, forcing the CSM to constantly fight to preserve its ‘space’ and the CFS as a whole.

To summarise the third condition for politicizing participation, the CFS has given priority voice to representatives of the organizations representing those most affected by hunger and food insecurity through what we called the ‘most-affected principle’. By recognizing the right of the CSM to autonomously self-organize, the CFS has secured the possibility for everyone, including ‘extremists’, to have the right to speak. In the case of the CFS, these extremists are social movements representing food producers and other civil society actors defending the right to food and food sovereignty. Following the theory, for a process to be political, those at the extremes need to be included. This is in opposition to many MSPs, which conform to an anti-political condition that encourages people to participate in ways that demonstrate a high degree of self-discipline (i.e., working towards consensus, making ‘relevant’ interventions). Those actors who show this self-discipline are often rewarded by being invited back, or invited to participate in related processes. In the case of the CFS, the autonomous nature of the CSM helps to ensure that more extreme voices from civil society are present and heard at the CFS.

## Challenges to the re-politicization of food security governance

We have argued above that politicizing food security governance is fundamental to just and sustainable food futures. We have also outlined, in line with the theory, how the CFS has implemented conditions that enable its politicization, notably through the participation of social movement actors representing the most affected. However, we note that across the literature there is a paucity of empirical assessment as to what happens once a space is politicized. Our research shows that politicization of the CFS has been met with strong resistance by those more invested in the maintenance of the status quo. In what follows we show how across the work of the CFS, there are multiple, and worrying examples of actors seeking to undermine or de-politicize the Committee. In the face of a politicizing CFS, there have been deliberate efforts by traditionally powerful actors to ‘reform the reform’ such as: delegations like the United States or Russia, trying to block topics from being discussed (for example agroecology (see below) and human rights respectively); Canada and other G7 countries challenging to normative basis of the CFS while also trying to limit the CFS to a niche role rather than as a global convergence role; and, reducing the policy making role of the CFS by promoting an exchange of best practices over policy outcomes (CSM 2017, 5–6). In what follows we focus on two examples that emerge clearly from our data: efforts to undermine the most-affected principle; and attempts to reduce the possibility of disagreements in policy negotiations on contentious issues.

### Challenging the most-affected principle

In recent years, the reform commitment to ensuring that the voices of those most affected by food insecurity are heard has been put under strong pressure, with implications for politics at the CFS. One of the direct causes of this shift has been the rapid development of the Private Sector Mechanism (PSM). Using its growing influence, the PSM has sought to obtain changes in the participation structure of the CFS in ways that threaten the prioritization of civil society voices (i.e. the most affected) within the CFS. Most notably, at CFS 43 (2016), the PSM sought parity with the CSM in terms of the number of seats on the Advisory Group, arguing that it should have ‘equal voice’ at the table (Bester et al. 2017). These efforts were supported by countries like the United States and Australia.

In the same year, the World Farmers’ Organization^4^ (WFO) also entered the scene, bringing some 40 representatives to CFS 43 (Schramm 2017). The WFO is an international association of farmers which represents ‘nano, small, medium and large-scale farmers’ and advocates ‘on behalf of farmers in global policy forums’ in an effort to ‘create the conditions for the adoption of policies aimed to improve the economic environment and livelihood of producers, their families, and rural communities’ (WFO 2017). Although the WFO was granted observer status and not participant status (like the CSM and PSM), the CFS Chair repeatedly granted the WFO speaking time in the CFS 43 Plenary, a clear challenge to the dynamics of participation outlined in the reform document. The presence of the WFO further added to existing dynamics of contest over representation and distribution of seats in the CFS Advisory Group. Again, directly challenging the balance of participation of the CFS reform, the WFO, backed by the PSM, advocated for the creation of a farmers’ mechanism (like the existing CSM and PSM), arguing that farmers were not adequately represented in the CFS. For civil society actors, the inclusion of the WFO as a participant in the CSM is a direct attempt to re-balance power in favor of the private sector. However, both the PSM and WFO backed down following the 2017 independent evaluation of the CFS, which dismissed the WFO’s call for participant status outside of the Private or Civil Society Mechanisms. Evaluators did not agree that farmers were not well represented in the CFS ‘as there are farmers in both [the civil society and private sector] mechanisms’ (Bester et al. 2017, xix). Despite this controversy, the WFO nevertheless secured ad hoc status in the CFS Advisory Group, shifting the balance of power in the Advisory Group back in favor of those who have traditionally held power.

The challenge to the most-affected principle does not only come from private sector actors. Based on interviews with member governments we note increased concern and resistance about the role of the CSM on the Advisory Group. Reflecting the influence of civil society actors vis-à-vis states, one state representative stated:

Basically, there’s no space for the Bureau to decide… like the Bureau needs to say, ‘Oh, okay, everything has been negotiated in the Advisory Group meeting, so now we just say yes.’ That’s not true you know. The Bureau’s decision right should be more (Interview 53).

Here the representative worries that participation of non-state actors, particularly civil society actors, is reducing the ability of states to take decisions in the Bureau.

Not all states representatives interviewed shared these concerns, but they did recognize the changing dynamics. For example, a state representative told us:

I hear a lot of member states arguing that the essence of the CFS is its intergovernmental nature because they see so many problems with this multi-stakeholder approach. I believe there’s so much value in it, and we have to come back to this, and really to convince also these member states that there is a value… not only to make it [the CFS] politically complicated but also to get something out of it (Interview 57).

Along these lines, we note a double trend that emerged through our interviews with governments. First, many states tend to overlook the most affected principle – which effectively reinforces the political nature of participation in the CFS—, in favor of the all-affected principle. They insist, for example, on giving the CSM and PSM equal consideration and feel that this equal consideration is key to the legitimacy of the CFS as a MSP. Second, many states perceive that the CSM does not fully participate in the diplomatic effort to find consensus and complain that the CSM defends its positions rather than seek compromise (Claeys and Duncan 2018b). While CSM actors contest this view, the frustration felt by state representatives reflects the discomfort around disagreement and the pressure facing civil society actors – the so-called extremists – to play along. In our view, attacks on the most affected principle are indicative of a process of de-politicization of the CFS as these attacks seek to limit the availability of spaces where the political can play out.

### Reducing the political in policy negotiations: The case of agroecology

The second challenge to politicization relates to how powerful actors seek to de-politicize the CFS by neutralizing debate through the application of a discourse of objectivity. We illustrate this trend by exploring the case of agroecology. Agroecology is a prime example of a political and contentious issue that managed to get onto the CFS agenda because of the participation rights of civil society.

Following the reform, agroecology was initially rejected as a policy gap for the CFS to possibly address (Duncan 2015, 198). In 2017, after a long process and a great deal of effort on the part of civil society, along with representatives from France and Switzerland, agroecology was included in the CFS’s Program of Work. As one key actor in the CSM explained:

The CFS sometimes wants to shy away from topics that are controversial, that could create not an easy consensus, that could create a political debate on different models and alternatives. …We spent like four meetings only trying to say that the CFS *could* have a discussion on agroecology. Not even saying what agroecology is but only saying, ‘Can we debate about agroecology here?’ We took four meetings extra just to say yes (Interview 36).

While agroecology recently made it onto the FAO’s agenda with a series of regional and international symposia, it remains contentious for many governments insofar as agroecology, as a science, movement and practice, seeks to make farming more socially just and environmentally sustainable (Wezel et al. 2009). Although these ambitions are not immediately controversial, it is the rejection of the global corporate food system that is often at the core of agroecological approaches (Levidow et al. 2014; Pimbert 2015; Rosset et al. 2017) that threatens the status quo. As Rosset et al. (2017, 1) state, ‘[w]hile some may wish to deny this, agroecology has a strong political element that is inseparable from its technical-biological aspect’. It is thus not surprising that most G20 countries were inactive in the FAO symposia and actively fought against having agroecology on the CFS agenda.

The tensions around including agroecology on the CFS agenda can be seen in the comments of one state representative:

We will have a workstream on agroecology. Very interesting, but was it so necessary? Because there are other fora, including FAO, which deal with agroecology. Was it so necessary to focus on agroecology? Because it’s an option for agriculture. It’s not really an issue linked to food insecurity. That’s my perception. They say, ‘Agroecology is a combat against the current global food system.’. … No. It’s not to combat the world food system (Interview 52).

The representative does not recognize the link between food security and agroecology, and dismisses the narrative that agroecology presents a valid alternative for food production and is therefore relevant for food security policy.

A review of the process leading towards negotiations on agroecology is instructive for understanding how the CFS makes space for competing visions and how some actors try to limit the political nature of these negotiations. The process of defining the draft scope of the independent HLPE’s report on agroecology, for example, uncovers a number of instances where the de-politicization process is made visible through the actions of and tools used by traditionally powerful actors (in this case, notably the United States and the Private Sector).

The draft scope for the HLPE report was released online for public online consultation in October 2017. In response to the draft scope, the Private Sector Mechanism (PSM) submitted a statement that encouraged ‘the HLPE as the source of scientific advice for CFS to focus on the scientific definition rather than the political movements and ideological debates’ (HLPE 2017a, 194). The reduction of debates to scientific definitions and expertise is a key strategy of depoliticisation. This de-politicized environment corresponds with a lack of negotiation and deliberation between different lived experiences and perspectives, as well a narrow view on (scientific) evidence. As a result, governance outcomes tend to codify or entrench hegemonic—predominantly neoliberal—forms of knowledge and reinforce elite power relations (Hughes et al. 2018; Clarke 2011). The United States’ response continued in a vein similar to that of the PSM. In their comment sent in by a representative from the Department of Agriculture, the United States noted the importance that:

this report offer a balanced assessment that does not pre-judge the contributions and limitations of various approaches or frame them in opposition to one another. Agroecological approaches and other innovations can be complementary, and the report should be focused on best practices for improving food security and nutrition, not pitting approaches against one another (HLPE 2017a, 163).

This statement from the United States functions to frame the structure of the proposed report as un-balanced, serving to delegitimize its scientific value. One representative from a UN agency active in the CFS noted that in response to these efforts, he was:

Disappointed, but maybe not completely surprised. It was a very odd-- At one stage it looks like it was going to be agroecology and – What were they calling it? It was going to be agroecology and bioengineering or something. It was something ridiculous that was the opposite of agroecology. I think CSM managed to advocate and successfully bring it back to agroecology (Interview 60).

This quote illustrates how important the participation of the CSM was to ensure that agroecology stayed on the CFS agenda in the face of the opposition of traditionally powerful actors.

The case of agroecology is indicative of a contentious issue being de-politicized by efforts to limit the political scope of a scientific report that has a mandate to explore diverse forms of knowledge. To eliminate the political, hegemonic actors such as the PSM, but also states, reject the normative elements of food security and the multiple (including political) dimensions of agroecology. As the quotes above illustrate, these efforts also involve disregarding civil society statements as ‘ideological’. As one diplomat told us: ‘I find sometimes that the CSM stifles debate by having too much of a predetermined ideological view on things’ (Interview 54). This sentiment was frequently repeated in interviews with other state representatives. This is not something that has escaped CSM actors. As one interviewee noted:

I think we’ve made gains on agroecology, partly because we have such a strong conviction underpinning us in the food sovereignty approach, which makes it easy. The downside of that is it makes it easy for us to become ideological, with the value of propositions they’re based on strong values that food sovereignty dictates. It’s at the heart of what we do. But they can use that to block us. They can claim that this is ideological. We hear that a lot from governments. I think it’s a strategic issue. How can we further our CSM objective while moving forward on the practical things... and not create an easy way for them to block us, by saying ‘oh, its ideological’ (Interview 31).

In response to this, and echoing Rancière’s definition of disagreement being about power and not misunderstanding, another actor in the CSM stated:

Those who call us ideological are mostly those who don’t like our positions. I don’t think is necessarily a problem of understanding. They understand mostly our positions but they don’t like them. For example, we are called ideological when fighting for human rights, women’s rights, the right to food… It is not like we take this as a light issue where we can give up (Interview 6).

This quote also reiterates concerns around self-discipline of stakeholders in MSPs and the challenge of maintaining so-called extreme views.

A discourse of objectivity or scientific rationality is often adopted by actors to limit or resist disagreement and maintain status quo. This has been used to reduce the scope of the agroecology debate in the CFS, for example, and is more widely made visible in calls for ‘evidence-based policies’ (Head 2010; Swyngedouw 2010). Yet a political policy process is one which recognizes not only a diversity of stakeholder views, but also reflects a diversity of forms of knowledge. We have shown that within the CFS there are active efforts to limit the diversity of perspectives and to reframe disagreement as ideological so as to delegitimize alternatives. So far, thanks to the strength and commitment of civil society actors in the CFS, and the politicizing mechanisms they have put in place within the CSM, these efforts have not been overly successful.

## Conclusions

In this paper we have interrogated a trend in the de-politicization literature that suggests enhanced participation of stakeholders, particularly through MSPs, restricts politics (Tsouvalis and Waterton 2011). As scholar-activists concerned about this trend, we have sought to explore the conditions or mechanisms that enable the politicization of MSPs. Towards this end we have used the CFS as case study of a governance space where these depoliticizing tendencies are being challenged, particularly by civil society actors. We have discussed three conditions that have helped politicize the CFS: a common set of rules of engagement outlined by the CFS reform process; representation of a diversity of views and forms of knowledge; and, protecting the rights of all actors to speak and be heard (Rancière 1998), particularly those most affected. We have further shown that while these rules and mechanisms operate as enabling conditions that support politicization, civil society actors also play a fundamental role. Indeed, the politicization of the CFS is directly linked to the politicized nature of the Civil Society Mechanism itself. Not only has the CSM gained the right to function as an autonomous and self-organized space, it has also assigned leadership roles to social movement representatives^5^ through a complex and sophisticated governance mechanism grounded in constituencies and quotas (Claeys and Duncan 2018a; Duncan 2016). We have outlined how the CSM has adopted its own set of political practices wherein it makes a clear distinction between constituencies from social movements representing affected groups from other secondary, or non-affected constituencies (such as NGOs). In so doing it maintains political roles for the former, and assigns support roles for the latter (Claeys and Duncan 2018a) and reinforces the most affected principle.

Beyond highlighting some necessary conditions for politicizing MSPs, another contribution of this paper has been to document the opposition that comes from efforts to politicize policy processes, particularly by traditionally powerful actors. Although the right of everyone to speak has been widely respected at CFS to date, it is coming under increasing attack by hegemonic actors (notably the United States and the Private Sector Mechanism). Given that an explicit objective of politicization is to open up pathways for counter-hegemonic possibilities (Moragues-Faus 2017; Mouffe 2005), this is not surprising. We could even consider it a reliable indication that politicization has indeed taken place at the CFS since the reform. Yet, in the face of a politicized CFS, there have been, and continue to be, deliberate efforts by powerful actors to ‘reform the reform’ such as: challenging the principle of the most affected, and neutralizing debate on contentious issues where there is fundamental disagreement. The political nature of the CFS could thus be the factor that leads to its decline if, for example, powerful actors shift attention and participation to other fora (so-called forum-shifting (Margulis 2015)). Indeed, alternative global food security governance spaces exist that are less prone to disagreement, and where states are held to lesser account due to limited participation of non-state actors, or more disciplined actors. In the face of these challenges, we argue for the importance of ensuring and reinforcing mechanisms that deliberately politicize participation in the CFS.

Our analysis of a politicizing CFS has highlighted some of these mechanisms. One is clearly distinguishing between different categories of stakeholders. The CFS reform distinguishes between states and CFS participants, retaining political decision-making with states (in case there is no consensus) while ensuring a diverse range of views inform and contribute to policy outcomes. Another mechanism is clearly distinguishing among different categories of non-state actors. The reform sets clear boundaries between CFS participants such as the CSM and the PSM, clearly un-levelling participation in favor of those most affected and traditionally excluded. The reform further ensures the autonomy and self-organization of civil society participants, which enables them to internally adopt political practices. While these mechanisms are a step in the right direction, they are at once insufficient and under threat, as efforts to challenge the most affected principles indicate.

Just as the de-politicization of food governance leads to a lock-in of neoliberal norms and values, often at the expense of alternatives, re-building sustainable and just food systems requires alternative visions that can only be made visible in politicized policy processes. The politicization of food governance is therefore fundamental to re-building food systems as it makes counter-hegemonic positions both visible and possible while re-invigorating policy processes through meaningful participation and engagement with contentious issues. Thus, while our analysis of the CFS has shown that the re-politicization of a policy space is met with active resistance and concerted efforts to maintain status quo, it is clear that to rebuild food systems, business as usual is not an option (IAASTD 2009).

## Footnotes

1 The UN’s own reporting on progress towards achieving this goal shows that, despite the need for increased investment in food security and agriculture, indicator 2.a.1 of the SDGs, the global agriculture orientation index – defined as the agriculture share of Government Expenditures, divided by the agriculture share of GDP – fell from 0.38 in 2001 to 0.24 in 2013 and to 0.21 in 2015 (UN 2017, 4).

2 It is important to acknowledge that the CSM and PSM are spaces wherein struggles over politics and politicization are also at play. For a review of the internal political dynamics of the CSM see Claeys and Duncan (2018a).

3 The biannual composition of the Advisory Group for 2016–17 was: FAO (1 seat), WFP (1 seat), IFAD (1 seat), Special Rapporteur on the right to food (1 seat), UN High-Level Task Force on the Global Food and Nutrition Security (1 seat), UN Standing Committee on Nutrition (1 seat), World Bank (1 seat), Civil Society Mechanism (4 seats), Private Sector Mechanism (1 seat), CGIAR (1 seat), Bill & Melinda Gates Foundation (1 seat) as well as two Ad hoc seats for WHO (1 seat) and World Farmers Organization (1 seat).

4 The WFO represents a range of farmers organizations – and the WFO is careful to have small-scale producers from the Global South speak on behalf of the organization at the CFS Plenary (observations CFS43 (2016) – but many of its members are made up of large-scale commercial producers.

5 We note that many participants in the CSM have rejected language around multi-stakeholder processes, calling instead for multi-actor approaches. In their view, multi-stakeholder governance wrongly suggests that all actors (e.g., states, civil society organizations and private sector) have the same rights to participation and the same stakes. They prefer the term multi-actor governance, which they claim highlights that while all actors have the right to discuss and contribute, only states hold decision-making power (Claeys and Duncan 2018b).
